# Self-Care Experiences of Individuals with Heart Failure: A Systematic Review and Metasynthesis

**DOI:** 10.3390/jcm15176723

**Published:** 2026-08-29

**Authors:** Israr Ahmad, Agata Bielecka-Dabrowa, Brunilda Subashi, Yi Chen

**Affiliations:** 1 Department of Biomedicine and Prevention, University of Rome Tor Vergata, 00133 Rome, Italy; israrahmadd008@gmail.com (I.A.); brunilda.subashi@univlora.edu.al (B.S.); chenyimiley@foxmail.com (Y.C.); 2 Department of Nursing, CECOS University of IT & Emerging Sciences, Peshawar 25000, Pakistan; 3 Department of Cardiology and Congenital Heart Diseases of Adults, Polish Mother’s Memorial Hospital Research Institute, 93-338 Lodz, Poland; 4 Department of Preventive Cardiology and Lipidology, Medical University of Lodz, 90-752 Lodz, Poland; 5 Faculty of Health, University of Vlora ‘Ismail Qemali’, 9400 Vlore, Albania

**Keywords:** self-care, self-management, experiences, individuals, heart failure

## Abstract

**Background:** Heart failure (HF) is a complex, life-threatening syndrome associated with substantial morbidity and mortality, reduced functional capacity, diminished quality of life, and considerable healthcare costs. Globally, HF affects more than 64 million individuals. Chronic heart failure (CHF) imposes a significant economic and personal burden on individuals, society, and the healthcare system, and places substantial demands on effective self-care. **Objective:** The purpose of this metasynthesis was to explore the self-care experiences of individuals with heart failure and the ways in which they manage their self-care. **Methods:** A comprehensive search was conducted from January 2014 to July 2025 across four major databases: PubMed/MEDLINE, Web of Science (Clarivate), Scopus, and ProQuest, and two gray-literature sources, OpenGrey and OAIster. A total of 320 records were exported to the Rayyan Systematic Review Management Platform, and after the screening process, only 19 were included for data extraction. The quality of included studies was assessed, and the data were synthesized using thematic synthesis. **Results:** Three overarching analytical themes were generated that capture multifaceted experiences of individuals living with heart failure and engaging in self-care. These included: adaptive and technology-supported self-management in daily life, emotional and psychological dimensions of self-care, and social, cultural, and environmental influences on self-care. **Conclusion:** Heart failure self-care is a dynamic and multifaceted process shaped by adaptive daily practices, emotional and psychological experiences, and social and cultural contexts. Individuals continuously adjust behaviors, manage symptoms through lived experience, and rely on supportive relationships and resources to sustain self-care. Recognizing this complexity is essential for developing individual-centered, culturally sensitive interventions that align with the lived realities of individuals with heart failure.

## 1. Introduction

Heart failure (HF) is a complex, progressive clinical syndrome characterized by impaired cardiac function and systemic congestion, affecting over 64 million individuals worldwide and contributing to substantial morbidity, frequent hospitalizations, and high mortality rates [1,2]. The management of HF requires both evidence-based medical interventions and active individual engagement in self-care behaviors, which are essential to maintaining clinical stability and improving outcomes [3,4].

Self-care in HF is a situation-specific process involving daily behaviors to maintain physiological stability, recognize symptoms, make timely decisions, and respond appropriately to changes in clinical status [5,6,7]. According to the situation-specific theory of self-care, self-care comprises three interrelated domains: self-care maintenance (routine actions to preserve health), self-care management (responding to emerging symptoms), and self-care confidence (self-efficacy to perform these behaviors) [5,8,9]. These domains are commonly assessed using validated instruments such as the Self-Care of Heart Failure Index (SCHFI), which provides a measure of individuals’ competence in self-care and predicts clinical outcomes [5,6,7,8,9].

Evidence consistently demonstrates that effective self-care is associated with reduced hospitalizations, lower mortality, improved quality of life, and better symptom control [8,10,11]. Despite this, many individuals with HF exhibit suboptimal self-care, with deficiencies across maintenance, management, and confidence domains, even when guideline-directed education is provided [12]. Qualitative evidence indicates that many individuals neglect essential self-care behaviors due to psychological, cultural, and motivational barriers, highlighting the challenge of translating guidelines into effective self-management [1,13].

Determinants of HF self-care are multifactorial, encompassing individual factors (e.g., knowledge, health literacy, emotional well-being), interpersonal support (e.g., caregiver involvement), system level factors (e.g., access to structured education and follow-up). Interventions such as telemonitoring, eHealth programs, traditional care models, and nurse-led education have shown promise in promoting self-care behaviors, though the comparative effectiveness and optimal strategies remain unclear [4,8,14]. A metasynthesis is conducted to synthesize individuals’ lived experience across studies, uncover common barriers and facilitators of heart failure self-care, and generate higher-order insights that cannot be captured by quantitative research alone. This approach informs theory development and the design of individual-centered interventions by integrating the nuanced perspectives of individuals [15,16].

Therefore, the purpose of this metasynthesis was to aggregate, interpret, and synthesize findings from qualitative studies examining the self-care experiences of individuals with heart failure. The review seeks to answer a central question: What are the self-care experiences and management strategies of individuals living with heart failure? By integrating findings across diverse qualitative studies, this metasynthesis advances understanding of the complex processes underpinning self-care in HF and provides evidence to guide the development of patient-centered interventions aimed at improving outcomes and quality of life.

## 2. Methods

### 2.1. Design

This systematic review used a metasynthesis design to integrate and interpret evidence from qualitative studies examining self-care and lived experiences of individuals with heart failure. Metasynthesis was chosen to move beyond descriptive aggregation by generating higher-level interpretive insights that are not apparent in single studies [17,18,19,20,21,22]. The protocol was registered in the PROSPERO database (CRD420251035982). The PRISMA 2020 checklist was used to guide reporting in this metasynthesis, incorporating updated recommendations that reflect current advances in identifying, selecting, appraising, and synthesizing studies. See Table S1 PRISMA 2020 checklist [23].

### 2.2. Literature Search

A comprehensive literature search was conducted from January 2014 to July 2025 across four major databases: PubMed/MEDLINE, Web of Science (Clarivate), Scopus, and ProQuest Central and two gray-literature sources, OpenGrey and OAIster. This metasynthesis was restricted to studies published within the specified period to ensure alignment with contemporary heart failure care. This period captures key developments influencing self-care frameworks and the integration of digital health technologies, all of which shape how individuals perceive and manage symptoms. Restricting the timeframe enhances conceptual consistency across studies and strengthens the relevance of findings to current practice [24,25]. Only studies published in English were included. This decision was guided by methodological considerations specific to qualitative studies, where preservation of contextual meaning, cultural expressions, and interpretative depth is critical [26]. Translation of qualitative research may alter nuanced meaning and affect interpretation of self-care experiences. Given that this review employed an interpretive approach to synthesize lived experiences, even minor alterations in meaning during translation could influence theme development and conceptual interpretations [27]. Furthermore, ensuring conceptual equivalence across translated qualitative data requires rigorous linguistic validation, which is essential but may introduce additional layers of interpretation [28]. Although addressing language inequities in science remains important, evidence suggests that language restrictions have minimal impact on the conclusions of systematic reviews. However, previous methodological research indicates that, particularly in qualitative evidence synthesis, language restrictions do not necessarily lead to significant differences in overarching themes or conclusions [29,30,31]. The language limitation is acknowledged in the strengths and limitations section [32,33]. The keywords and MeSH words were ‘self-care’, ‘self-management’, ‘heart failure’, ‘behavior’, ‘health risk behavior’, ‘in-hospital’, ‘quality of life’, ‘mortality’, ‘rehospitalization’, ‘health care costs’, ‘qualitative research’.

*Search strategy*: The keywords were (self-care OR self-management) AND heart failure AND (behavior OR health risk behaviors) AND (in-hospital days OR quality of life OR mortality OR rehospitalization OR health care costs) AND qualitative research. Filters: from 2014 to 2025. Additional details are available in the Supplementary Table S2, Full search strategy. Outcome-related terms were included to capture studies examining broader consequences of self-care, including quality of life, rehospitalization, mortality, and healthcare utilization, consistent with the review objectives.

*Results displayed*: The database search identified 59 results in PubMed, 41 in Web of Science, 120 in Scopus, and 100 in ProQuest Central. The gray-literature search produced no results in either OAIster or OpenGrey. The Boolean variables “AND” and “OR” were used during the literature search as required.

### 2.3. Study Selection

**Figure 1:**
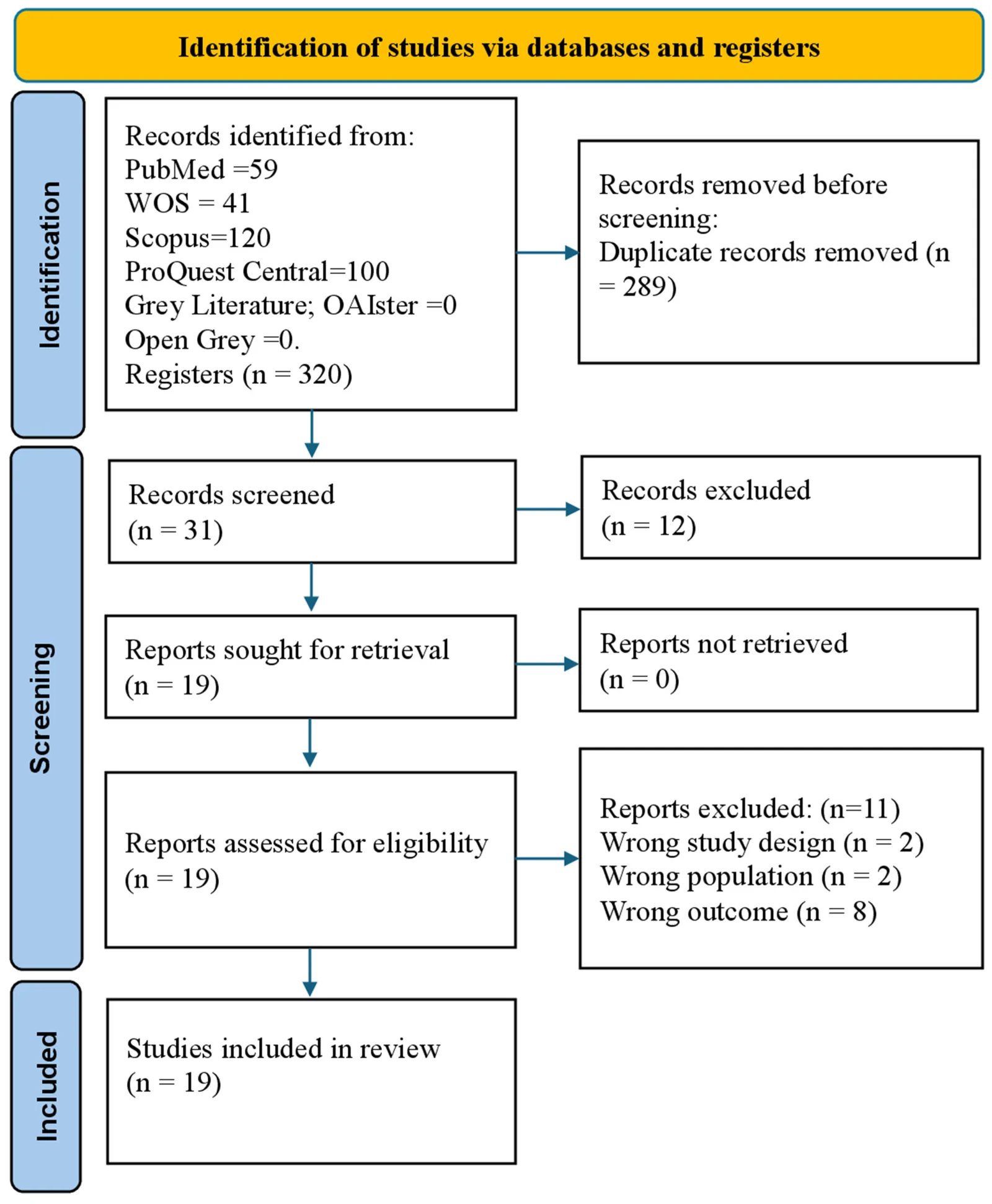
Flow chart for included studies.

The study selection process is illustrated in the PRISMA flow diagram. A total of 320 records were identified through electronic databases and registers and imported into the Rayyan Systematic Review Management Platform Rayyan Systems, Inc. Cambridge, MA, USA. After removing 289 duplicates, 31 records were screened at the title and abstract level, of which 12 were excluded due to inappropriate study design (*n* = 2), irrelevant population (*n* = 2), or non-eligible outcome (*n* = 8). The remaining 19 full texts were assessed for eligibility. Ultimately, 19 studies were included in the final systematic review. The two independent reviewers (IA & BS) completed the initial and full-text screening, and discrepancies were resolved with the third reviewer (YC). A list of excluded full-text studies and reasons for exclusion is provided in Supplementary Table S3, List of excluded studies. This process has been summarized in Figure 1 using PRISMA guidelines 2020 [23].

### 2.4. Population and Context

The review includes adults with heart failure from diverse global regions, encompassing both men and women. Studies explored individuals’ experiences of self-care, highlighting how cultural, social, and environmental factors, as well as family support and access to healthcare or digital resources, shaped self-care practices.

### 2.5. Study Inclusion and Exclusion

The focus of this study was on individuals with HF engaging in self-care. The inclusion criteria were as follows: (a) sources published in English language from January, 2014 to July 2025, (b) peer review articles such as original research articles, (c) sources that explored the self-care in heart failure individuals from their perspectives, (d) sources related to any setting (in hospital or outpatient setting), (e) sources with qualitative research and qualitative part of mixed methods studies addressing self-care in heart failure individuals, (f) age ≥ 18 years. The exclusion criteria are (a) original qualitative studies and mixed methods studies including individuals with heart failure along with informal caregivers and/or health care professionals’ perspectives, (b) quantitative studies about individuals with heart failure, and (c) literature reviews, discussion papers, dissertations, commentaries, editorials, and opinions about the current topic under study.

### 2.6. Critical Appraisal

A critical appraisal checklist, the ‘Critical Appraisal Skills Program (CASP),’ was utilized for appraising the selected studies presented in critical appraisal Table 1. This checklist contains ten questions with three main areas: (a) Are the results of the study valid? (b) What are the results? (c) Will the results help locally [34]? Each item in CASP is scored based on three options: ‘Yes’, ‘Can’t Tell’, and ‘No’. Although the CASP does not provide a formal numerical scoring system, a pragmatic threshold of ≥7 “Yes” responses was adopted for this review to support consistent appraisal. This threshold represents a review-specific adaptation and is not a validated CASP criterion. Studies with ≥7 " Yes " responses were considered high quality, while fewer than 7 “Yes” responses were considered low quality. The methodological quality of included studies was independently assessed by two reviewers (IA and BS) using a standardized critical appraisal tool. To ensure consistency, the appraisal tool was initially applied to a sample of 10 studies, and discrepancies were discussed to achieve shared understanding of the assessment criteria. Any conflicts arising and discrepancies were resolved through discussion until consensus was achieved, and quality scores were updated accordingly. Studies were not excluded based on quality, as even those with lower ratings can provide valuable contextual insights [20]. During synthesis, greater analytical weight was given to findings derived from studies assessed as higher quality, while results from the lower-quality studies were used to support the interpretations drawn from high-quality evidence.

### 2.7. Data Extraction and Charting

Two reviewers (IA & BS) independently performed data extraction using the ‘Data Extraction Checklist’, which is displayed in Table 2. The most recent guidelines were followed for preparing the summary tables for this metasynthesis [54]. Each reviewer independently collected the required information and then cross-checked their findings before finalizing the complete extraction sheet. The data extraction sheet contained information about the authors, country, year, purpose, definitions, methods (design, sample, sample size, sampling technique, data collection, and data analysis). During the extraction process, any disagreements between the reviewers were addressed through discussion with the involvement of a third reviewer (YC).

### 2.8. Analysis and Synthesis

The thematic synthesis approach was followed for synthesis of findings [55]. The thematic synthesis consists of three main steps: (a) coding the text line by line; (b) the development of descriptive themes; and (c) generation of analytical themes. Original studies were reviewed for participants’ quotes and findings for theme extraction and review.

First, line-by-line reading was completed for the findings and discussion sections. Second, two authors (IA & BS) independently read selected articles multiple times to develop a comprehensive understanding of the findings and coded all the relevant findings. The codes comprise words and phrases used in participants’ quotes, the description of qualitative themes and sub-themes presented in the reviewed studies, and the interpretations of study authors. Third, codes were organized into first-order themes and then collated into descriptive themes. During this stage of analysis, two researchers (IA & BS) worked together and moved back and forth between the codes and studies and revised any discordant descriptive themes. Finally, the descriptive themes were collated based on their shared meaning and converted into analytical themes. The line-by-line codes were kept close to the findings of the studies, but personal interpretations were incorporated during the generation of analytical themes. This was performed to make our analysis more interpretative than descriptive.

## 3. Findings

### 3.1. Study Characteristics

The studies were conducted across multiple countries, with the majority based in the United States (USA) (*n* = 7). Additional studies were carried out in Europe (United Kingdom/England, Ireland, Italy, Germany, the Netherlands; including one multi-country European study), Canada, Australia, and several Asian countries (South Korea, Singapore, Thailand). One study was conducted in Turkey, representing a low-income setting. Overall, the evidence reflects a predominance of high-income Western countries, with more limited representation from low- and middle-income regions. Detailed information is presented in Data Extraction Table 2.

#### Themes

**Figure 2:**
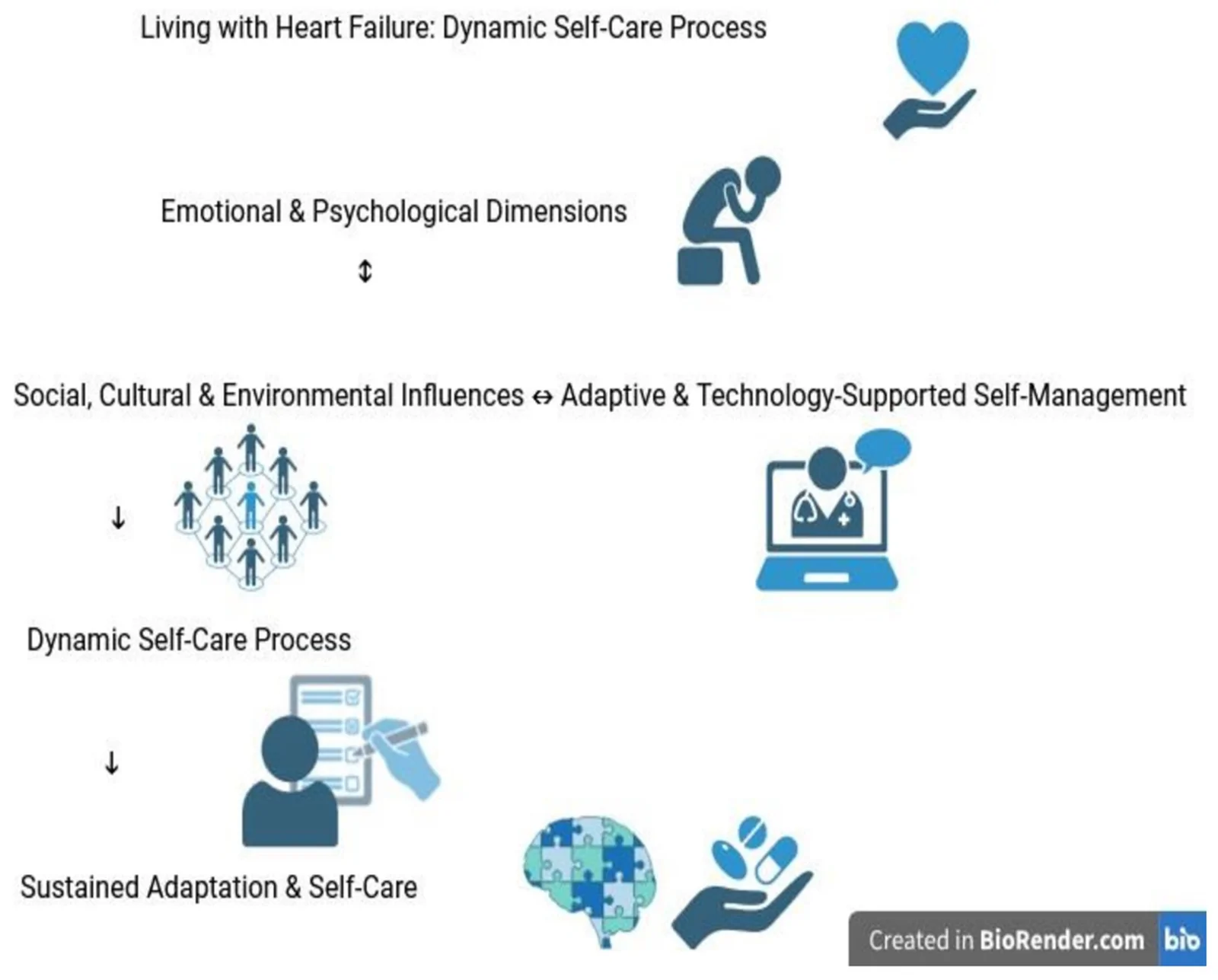
Visual representation of analytical themes.

The metasynthesis generated three overarching analytical themes that capture multifaceted experiences of individuals living with heart failure and engaging in self-care. These included: adaptive and technology-supported self-management in daily life, emotional and psychological dimensions of self-care, and social, cultural, and environmental influences on self-care. A summary of the themes and codes is given in thematic synthesis Table 3 and conceptual diagram summarizing the analytical themes (Figure 2).

### 3.2. Analytical Theme 1: Adaptive and Technology-Supported Self-Management in Daily Life

The first analytical theme consisted of six descriptive themes: (a) recognizing and responding to symptoms, (b) activity, environment, and routine modification and monitoring, (c) adjusting behavior, (d) coping with challenges and temptations, (e) technology as a self-care facilitator, and (f) barriers to technology use. Several studies support this theme [35,36,37,38,40,43,44,45,47,48,49,50,51,52]. This analytical theme highlighted how individuals with heart failure navigate the complexity of daily life by continually adjusting behaviors, routines, and environments. Their self-care efforts demonstrate a practical, embodied knowledge built over time, combining experiential learning, cultural values, and selective technological support. Technology emerged as a contextual facilitator of adaptive self-care, supporting symptom monitoring, adherence, and communication, while its use was shaped by individuals’ capabilities, trust, and preferences. Adaptive strategies helped individuals maintain functional independence and emotional stability despite fluctuating symptoms. Technology played a growing but uneven role, offering new avenues for monitoring and adherence, yet shaped by digital literacy, trust, and interpersonal preferences.

### 3.3. Recognizing and Responding to Symptoms

Individuals develop a fine-tuned awareness of early bodily cues, which they interpret through personal experiences, cultural beliefs, and past episodes. This awareness was not merely cognitive; it was bodily intuitive and shaped by trial and error. Individuals described monitoring subtle changes in breathing, fatigue, chest sensations, or anxiety that signaled worsening symptoms. True self-care, for many, began with noticing these cues [35,36,37,38,40,43,44,45,47,48,49,50,51,52].

Once symptoms were recognized, individuals quickly used simple strategies such as changing position, slowing activity, or using comforting items to regain control. Anticipating symptoms became essential, as individuals learned to identify triggers and adjust their behavior proactively. Cultural beliefs also shaped how symptoms were interpreted and managed, with some viewing them through spiritual or moral lenses that guide their responses [35,36,37,38,40,43,44,45,47,48,49,50,51,52].

“I’m lying in bed and unable to sleep, then I start getting stressed. I’m uncom-fortable, my mind’s wandering all over the place and I just generally don’t like it…but if I come and sit down here [in the chair] and got my dressing gown, I’ve got a rug I put over my legs and feet and everything, then I am a lot more com-fortable and relaxed to lying in bed. (77-year-old male with hypertensive CHF NYHA IV)” (p. 15) [35].

### 3.4. Activity, Environment, and Routine Modification

Living with limited energy capacity requires constant evaluation of how to perform even simple tasks. Individuals adopted pacing, prioritization, and energy-conserving techniques, sometimes modifying lifelong habits. Activities are strategized rather than spontaneous, with individuals describing deliberate “calm” or “slow” approaches to avoid exhaustion [35,36,37,38,40,43,44,45,47,48,49,50,51,52].

Environmental modifications were essential, with supportive chairs, rugs, and adapted tools helping individuals maintain independence and reduce strain. Integrating culturally meaningful routines like gardening, religious practices, or household roles allowed individuals to preserve identity while accommodating physical limitations [35,36,37,38,40,43,44,45,47,48,49,50,51,52].

“[About] physical activity, I do a lot of things, but calmly. Calmly. And also with a sort of caution, because if you get tired it’s not good.” (P6) (p. 4) [44].

### 3.5. Monitoring and Adjusting Behavior

Individuals employed self-monitoring tools to track symptoms, medication adherence, and physiological measures. Behavioral adjustments were guided by real-time feedback from devices or observation of bodily responses. Confidence in using technology developed over time, enhancing self-efficacy. Individuals personalized routine-based monitoring outcomes. Acceptance of technological support facilitated engagement in preventive behavior. Adjustments of technological support facilitate engagement in preventive behaviors. Adjustments often require trial-and-error learning. Monitoring provided reassurance and guided decision-making [35,36,37,38,40,43,44,45,47,48,49,50,51,52].

“Very easy to use…very easy to use. I was a bit apprehensive at the start. Because as I said I am not a techie person, I’m not into gadgets or anything like that. A bit apprehensive, but it only took me a day or two to fall into line with it and find then my way around with it. I found it very useful. One thing led onto another and I find it very useful now. [Male, 68 years, inadequate self-care]” (p. 12) [45].

Individuals actively manage dietary, lifestyle, and behavior temptations to prevent symptom exacerbation. Risky behaviors were replaced with supportive alternatives, while occasional indulgence was balanced with corrective strategies. Adaptive approaches were personalized and integrated into daily routines. Individuals emphasized gradual lifestyle modifications over abrupt changes. Coping strategies reflected experiential learning and prioritization of health outcomes. Cultural and social norms influenced dietary choices. Flexibility in self-care supports long-term adherence [35,36,37,38,40,43,44,45,47,48,49,50,51,52].

“Now, for example, I work in the garden. Of course, I don’t work with a pickaxe. Be-cause digging is uncomfortable when working with a shovel. But when I work with the waist shovel, it is good. Now I make a garden, I plant in the ground there, here I plant veg-etables. Now, when I deal with these slowly, it doesn’t do anything to me. Then I feel better. (Can)” (p. 10) [49].

### 3.6. Technology as a Self-Care Facilitator

Digital tools provided reminders, tracking capabilities, and platforms for communication with healthcare providers. Technology facilitated adherence to medications, lifestyle modifications, and symptom monitoring. Individuals adopted tools to align with personal values and routines. Positive experiences with technology increased engagement and confidence. Individuals used technology to share data with healthcare providers for informed guidance. Integration of devices supports accountability and motivation. Cultural and personal preferences shaped adoption [35,36,37,38,40,43,44,45,47,48,49,50,51,52].

“Absolutely I would use it. Because it’s easier…It would help a whole lot. Because it would show [my physician] when or if I was adhering to the protocol. He’d know I’m taking my medicine or if I’m not. [Aged 59 years, male]” (p. 8) [41].

### 3.7. Barriers to Technology Use

Challenges included limited digital literacy, preference for in-person interactions, and skepticism about reliability. Individuals valued human interaction and personalized assessment over automated tools. Digital tools were seen as supplementary rather than a replacement for professional care. Individuals expressed caution about adopting technology without guidance. Barriers influenced engagement and adherence [35,36,37,38,40,43,44,45,47,48,49,50,51,52].

“From a patient’s point of view, there is nothing like a human looking at you. I have to say that. This artificial intelligence is great and there is a place for it, but you can’t replace the human, in my view.” (post, IRL, female, 50–59 years)” (p. 8) [40].

Analytical Theme 2: Emotional and Psychological Dimensions of Self-Care

The second analytical theme comprised two descriptive themes: (a) emotional impact of symptoms, and (b) identity and role changes. Several studies support this theme [35,36,37,38,39,41,42,47,49]. This theme reflects the profound emotional and psychological impact of heart failure on individuals’ self-care practices. Individuals experienced fear, anxiety, and uncertainty associated with symptom predictability and disease progression. Changes in autonomy, physical abilities, and social roles influenced identity and self-perception. Psychological resilience, coping strategies, and cultural interpretations shaped engagement in daily self-care. Emotional experiences were intertwined with symptom management, motivation, and overall well-being.

### 3.8. Emotional Impact of Symptoms

Heart failure symptoms elicited fear of deterioration and mortality, particularly during nocturnal episodes. Anxiety arose from the unpredictability and intensity of symptoms. Cultural and spiritual frameworks influenced emotional interpretation and coping strategies [35,36,37,38,39,41,42,47,49]. Emotional distress sometimes triggered increased vigilance in self-care. Individuals reported sleeplessness, rumination, and heightened worry. Awareness of symptom severity shaped prioritization of daily activities. Emotional experiences were closely linked to symptom monitoring. Psychological support mitigated distress and enhanced self-management [35,36,37,38,39,41,42,47,49].

“These times in the middle of the night when you’re really frightened…oh am I going to die now you know….because you’ve nothing else to do during the night, just lie there and think. (86-year-old female with hypertensive CHF NYHA III)” (p. 11) [35].

### 3.9. Identity and Role Changes

Heart failure led to reduced autonomy, limiting participation in meaningful social and family roles. Individuals experienced frustration, emotional distress, and a decline in social value. Inability to meet previous expectations generated feelings of inadequacy. Loss of roles affected personal identity and self-perception. Individuals described renegotiation of relationships and responsibilities. Role limitations impacted engagement in physical and social activities. Awareness of dependency influenced coping strategies. Acceptance and adaptation were necessary for psychological adjustments [35,36,37,38,39,41,42,47,49].

“My husband is incredibly active…So we can’t go for bike ride together [because] I’d go maybe 10 or 15 km, and he likes to go for 150. Never mind the speed. So we can’t do many sports together … we don’t do activities together, pretty much across the board. … I think that’s probably been the hardest thing that I’ve had with relationships: appearing normal and knowing that I can’t do what every-body else can do, or what I should be able to do if I was a healthy version of me. ID 17, [Caucasian], Female, 50 years old” (p. 4) [47].

### 3.10. Analytical Theme 3: Social, Cultural, and Environmental Influences on Self-Care

The third theme included four descriptive themes: (a) support and guidance from healthcare providers, (b) social support and companionship, (c) cultural contexts in self-care, and (d) motivation through emotional and cultural connections. Many studies included in the review support this theme [37,38,39,40,41,43,44,45,46,48,49,50,53]. This theme underscores the influence of social networks, cultural norms, and environmental contexts on self-behavior. Support from healthcare providers, family, and peers enhanced motivation, confidence, and adherence. Cultural values shaped dietary habits, activity patterns, and interpretations of illness. Environmental factors such as access to resources and culturally meaningful activities influenced daily management. Individuals integrated social, cultural, and emotional considerations into self-care practices.

### 3.11. Support and Guidance from Healthcare Providers

Professional guidance served as a trusted source of reassurance and motivation. Individuals relied on healthcare providers for validation of self-care strategies. Culturally sensitive and personalized advice enhanced adherence and confidence. Reassurance reduced anxiety and informed decision-making. Regular communication with healthcare providers strengthened accountability. Individuals integrated guidance into daily routines while adapting to personal circumstances [37,38,39,40,41,43,44,45,46,48,49,50,53].

“You look up to professional and you need that reassurance to say: ’what I am doing now, is not going to impact on my long-term health’; ’is it the right thing to do?’ and if the answer comes back from a professional you trust, and I have im-plicit trust, explicit trust as well, in my consultant…then if she says: ’that’s a good thing to do’, then I will doit.” (p. 11) [37].

### 3.12. Social Support and Companionship

Family, friends, and peers provided emotional reinforcement and practical assistance. Shared activities strengthened motivation and engagement in self-care. Emotional support helped manage stress and symptom-related anxiety. Individuals noted overprotective caregiving could limit independence. Social interactions enhanced adherence and fostered resilience. Reciprocal support nurtured a sense of belonging and well-being. Practical guidance complemented self-monitoring efforts [37,38,39,40,41,43,44,45,46,48,49,50,53].

“I rely on my wife and daughter for symptom management. Drug therapies are regularly followed by my wife” (p. 10) [48].

### 3.13. Cultural Contexts in Self-Care

Cultural norms influenced dietary habits, activity choices, and self-care strategies. Individuals balanced medical advice with traditional practices and social obligations. Festive and seasonal food practices posed challenges to adherence. Respect for elders and social expectations guided behaviors. Cultural framing shaped symptom interpretation and coping strategies. Individuals negotiated personal health priorities within broader social contexts. Self-care was embedded in culturally meaningful routines [37,38,39,40,41,43,44,45,46,48,49,50,53].

“I mean to be realistic, sometimes you want to be fast or you have work to do, then you naturally end up eating things that are not so healthy” (Sam, 35). “Because come back already very late. More tiring (if have to cook at home)”(William, 41).” (p. 7) [36].

### 3.14. Motivation Through Emotional and Cultural Connections

Individuals derive motivation from family responsibilities and cultural expectations. Protecting children’s well-being and supporting partners reinforced commitment to self-care. Engagement in preventive behaviors was linked to moral and social duties. Emotional connections strengthened adherence to treatment and lifestyle modifications. Motivation fluctuated with perceived benefits for self and others. Cultural and familial obligations shaped goal setting and prioritization. Individuals integrated personal and social motivations to maintain health [37,38,39,40,41,43,44,45,46,48,49,50,53].

“My worry is that if I were to go, my missus will be very sad. That’s the only thing that bugs me. Whatever keeps her happy I will try. So, she’s my main mo-tivation.” (Tom, 50). (p. 5) [36].

## 4. Discussion

The purpose of this metasynthesis was to aggregate, interpret, and synthesize findings from qualitative studies about experiences of individuals with heart failure regarding self-care. Grounded explicitly in the three analytical themes, the discussion situates these findings within the broader self-care literature while remaining focused on the experiential dimensions illuminated by the synthesis.

The first analytical theme positions self-care as an adaptive, experience-based process rather than a static set of prescribed behaviors. Consistent with the systematic reviews reporting suboptimal self-care levels among individuals living with heart failure, particularly in symptom monitoring and lifestyle modification, the present findings demonstrate that individuals actively learn to recognize and respond to bodily cues through trial-and-error and lived experience [1,12]. This experiential knowledge complements, and sometimes substitutes for, formal education, echoing prior qualitative synthesis that emphasizes symptom perception as the cornerstone of effective self-care [5,7].

Activity pacing, environmental modification, and routine restructuring emerged as an essential strategy for maintaining functional independence. These findings are aligned with the evidence that individuals prioritize energy conservation and role preservation over rigid adherence to clinical recommendations [4]. Rather than disengagement, such adaptations reflect intentional self-regulation aimed at sustaining daily life, reinforcing the view that self-care is highly contextual and individualized.

Technology functioned as both an enabler and a constraint within this adaptive process. Consistent with the meta-analysis demonstrating benefits of eHealth and telemonitoring interventions on adherence and symptom control [8,10,11], individuals described increased reassurance, accountability, and confidence when digital tools were perceived as usable and trustworthy. However, barriers related to digital literacy, preference for human interaction, and skepticism towards technology limited engagement. These findings resonate with umbrella and systematic reviews indicating that technology-supported self-care is most effective when embedded within relational care and tailored to individual capabilities [3,9]. Technology, therefore, should be understood as a supplementary component within adaptive self-care, not a replacement for professional or relational support.

The second analytical theme underscores the uncertainty of emotional and psychological experiences in shaping self-care behaviors. Fear, anxiety, and uncertainty, particularly during symptom exacerbations, were integral to how individuals monitored symptoms and prioritized activities. Disease-specific individual-reported outcome measures can complement clinical assessment by capturing individuals’ perspectives on symptom burden and self-care experiences [56]. These findings are consistent with reviews demonstrating a strong association between emotional distress, perceived symptom burden, and self-care engagement [2,15]. Emotional responses often heightened vigilance, suggesting that anxiety can function as both a barrier and a motivator for self-care, depending on coping resources and support.

Identity disruption and role changes further shaped self-care trajectories. Loss of autonomy and inability to fulfill culturally or socially valued roles negatively affected self-perception and motivation, echoing qualitative evidence that heart failure challenges personal identity and social participation [13,15]. The need to negotiate the roles and expectations required psychological adjustment, highlighting that self-care extends beyond behavior to encompass meaning-making and identity reconstruction. Positive psychological experiences such as acceptance and resilience have been shown to support adherence and engagement, reinforcing the importance of integrating emotional and psychological support into self-care interventions [16].

The third analytical theme emphasizes self-care as a socially and culturally situated practice. Support and reassurance for healthcare providers were central to confidence and sustained engagement, consistent with evidence that professional trust and culturally sensitive approaches improve outcomes.Guidance enhances adherence and self-care capacity [7,14]. Individuals did not passively follow advice; rather, they integrated professional recommendations into personal, social, and cultural contexts.

Family and social networks played a dual role, offering emotional reinforcement and practical assistance while sometimes constraining autonomy through overprotective behaviors. This aligns with systematic reviews identifying social support as both a facilitator and a potential limiter of self-care independence [4,6]. Importantly, motivation rooted in family responsibility and emotional connection emerged as a powerful driver of adherence, consistent with qualitative synthesis highlighting moral obligation and relational meaning as central to self-care motivation [7,15].

Cultural context may influence dietary practices, symptom interpretation, and lifestyle choices. Individuals navigated tensions between medical advice and cultural norms, particularly around food and social obligations. These findings reinforce calls for culturally responsive self-care interventions that acknowledge social expectations and lived realities rather than relying solely on standard education [1,4]. The limited representation of low-and middle-income countries may constrain understanding of diverse social and cultural influences on self-care, highlighting the need for contextually responsive approaches. Taken together, the three themes indicate that self-care in HF is a dynamic process of experiential adaptation rather than a simple implementation of prescribed behaviors. Experiential knowledge links symptoms and emotional experiences with self-care decision-making, while healthcare support, family relationships, culture, and technology shape how this knowledge is interpreted and applied. Thus, self-care involves continuous learning, interpretation, negotiation, and adaptation within everyday contexts.

The eight lower studies were interpreted cautiously due to methodological limitations; however, the consistency across the studies supports the identified patterns. This metasynthesis extends beyond the previous reviews [15] by conceptualizing self-care as an interactive, contextually negotiated process, with experiential knowledge shaping symptom interpretation, decision-making, and adaptive self-care. Compared with the previous qualitative synthesis [7,15], this metasynthesis conceptualizes self-care experiences in HF as a dynamic, contextually negotiated process shaped by experiential knowledge, emotional response and sociocultural and healthcare contexts.

### 4.1. Implications and Future Directions

This study underscores that improving outcomes in heart failure requires a paradigm shift from disease-centered management to truly patient-centered, contextually grounded care. Future research should move beyond symptom control to address the complex interaction between the physical, psychological, social, and cultural dimensions that shape self-care. Intervention development should evolve toward personalized, theory-driven models that integrate behavioral, motivational, and digital components to enhance sustainability and real-world impact. Digital health technologies show promise in supporting self-management; however, further investigation is required to assess long-term usability, equity of access, and seamless integration into clinical care pathways. Additionally, interventions that leverage positive psychological constructs, such as hope, resilience, and optimism, may enhance adherence and improve quality of life. Research should also prioritize culturally sensitive approaches to ensure applicability across diverse and underrepresented populations. Finally, adopting flexible, low-burden study designs and engaging individuals in the co-design of interventions is critical to maximizing participation, relevance, and the translational impact of research findings.

### 4.2. Strengths and Limitations

This metasynthesis provides a comprehensive and integrative understanding of the self-care experiences of individuals living with heart failure, drawing together evidence from diverse geographical, cultural, and healthcare contexts. The review followed systematic and transparent procedures in accordance with the PRISMA guideline, ensuring methodological rigor and credibility. By including both published and gray literature, it minimized publication bias and captured a wider range of perspectives. The use of thematic synthesis enabled the generation of higher-order analytical insights that extend beyond individual studies, offering meaningful conceptual and practical implications for clinical practice and intervention development.

Several limitations should be noted. The review was restricted to English-language publications published between 2014 and 2025, which may have excluded relevant earlier qualitative evidence and introduced selection bias, particularly as most included research was conducted in Western countries. Heterogeneity in clinical settings and populations may limit the transferability of the synthesized themes across HF contexts. As a secondary analysis, the synthesis was limited by the quality and contextual detail of the original publications. The underrepresentation of studies from low- and middle-income countries also limits global generalizability. Finally, although qualitative synthesis involves subjective interpretation, independent coding and consensus discussions were undertaken to enhance credibility.

## 5. Conclusions

Heart failure self-care is a dynamic and multifaceted process shaped by adaptive daily practices, emotional and psychological experiences, and social and cultural contexts. Individuals continuously adapt their behaviors, manage symptoms through lived experience, and rely on supportive relationships and available resources to sustain effective self-care. Recognizing this complexity is essential for developing individualized, culturally sensitive interventions that align with the lived realities of individuals with heart failure.

## Figures and Tables

**Table 1:** Critical appraisal.

	*Was There a Clear* *Statement of the Aims of the* *Research?*	*Is a Qualitative* *Methodology Appropriate?*	*Was the Research Design* *Appropriate to Address the* *Aims of the Research?*	*Was the Recruitment* *Strategy Appropriate to the* *Aims of the Research?*	*Was the Data Collected in* *a Way that Addressed the* *Research Issue?*	*Has the Relationship* *Between Researcher and* *Participants been* *Adequately Considered?*	*Have Ethical Issues been* *Taken into Consideration?*	*Was the Data Analysis* *Sufficiently Rigorous?*	*Is there a Clear Statement* *of Findings?*	*How Valuable Is the Research?*	*Total*
[35] (Walthall, Jenkinson, & Boulton, 2017)	Y	Y	Y	Y	Y	N	Y	C	Y	Y	High Quality
[36] (Chew, Sim, Cao, & Chair, 2019)	Y	Y	Y	Y	Y	Y	Y	C	Y	Y	High Quality
[37] (Amirova et al., 2022)	Y	Y	N	Y	Y	Y	Y	Y	Y	Y	High Quality
[38] (Ivynian et al., 2020)	Y	Y	Y	C	C	Y	Y	C	Y	Y	High Quality
[39] (Koontalay, Botti, & Hutchinson, 2024)	Y	Y	Y	C	Y	C	Y	Y	Y	Y	High Quality
[40] (Neumann et al., 2024)	Y	Y	N	Y	Y	Y	Y	Y	Y	Y	High Quality
[41] (Chai et al., 2024)	Y	C	N	Y	Y	N	Y	C	Y	Y	Low Quality
[42] (Dickens, Dickson, & Piano, 2019)	Y	C	C	Y	Y	Y	Y	N	Y	Y	Low Quality
[43] (Son, Oh, & Kim, 2020)	Y	Y	C	Y	Y	Y	Y	Y	Y	Y	High Quality
[44] (Trenta et al., 2022)	Y	Y	Y	Y	Y	Y	Y	Y	Y	Y	High Quality
[45] (Johnston et al., 2022)	Y	Y	C	C	Y	C	Y	Y	Y	Y	High Quality
[46] (Myers et al., 2020)	Y	Y	Y	Y	Y	Y	Y	Y	Y	Y	High Quality
[47] (Surikova et al., 2020)	Y	C	C	Y	Y	C	Y	Y	Y	Y	Low Quality
[48] (Guidotti et al., 2022)	Y	C	C	Y	Y	C	Y	C	Y	Y	Low Quality
[49] (Seckin et al., 2024)	Y	Y	Y	Y	Y	Y	Y	Y	Y	Y	High Quality
[50] (Radhakrishnan et al., 2020)	Y	C	Y	C	Y	C	Y	C	Y	Y	Low Quality
[51] (Celano et al., 2020)	Y	Y	Y	C	Y	C	Y	C	Y	Y	Low Quality
[52] (Hall et al., 2014)	Y	N	C	C	C	N	Y	N	Y	Y	Low Quality
[53] (Carroll, Hahn, & Grady, 2022)	Y	C	C	Y	Y	Y	C	C	Y	Y	Low Quality

Y = Yes, N = No, C = Cannot tell.

**Table 2:** Data extraction table.

Authors/Year/Country	Purpose	Reported Definitions in the Reviewed Literature	Methods
[35] (Walthall, Jenkinson, & Boulton, 2017) United Kingdom	To explore how patients with CHF describe their experiences of breathlessness, its pattern, impact on daily life, and self-management strategies.	Dyspnea, breathlessness	**Design:** Descriptive qualitative **Sample:** Patients with heart failure with reduced ejection fraction (HF-REF), mean age 72.7, 60% male. **Sample Size:** 25 **Sampling Technique:** Purposive **Data Collection:** Semi-structured interviews at participants’ homes (45–90 min) **Data Analysis:** Braun & Clarke’s 6-step thematic analysis
[36] (Chew, Sim, Cao, & Chair, 2019) Singapore	This study aimed to adopt the TST as a guiding framework to explore the underlying mechanism by which HF self-care behavior improves.	Self-care	**Design:** Descriptive qualitative **Sample:** Patients with heart failure, mean age 56.1, 76.5% male. **Sample size:** 17 **Sampling Technique:** Purposive **Data Collection:** Unstructured interviews conducted in a hospital setting (15–42 min) **Data Analysis:** Braun & Clarke’s 6-step thematic analysis
[37] (Amirova et al., 2022) United Kingdom	To expand the understanding of the barriers and enablers to physical activity in older adults (≥70 years old) living with HF. And to explore perceived clinical, environmental, and psychosocial barriers and enablers in older adults living with HF.	Not provided	**Design:** Descriptive qualitative design with phenomenological–hermeneutic approach **Sample:** 16 participants with a mean age of 79.19 (SD = 5.15), four of whom were women, and 12 were men. **Sample Size:** 16 **Sampling Technique:** Purposive **Data Collection:** One-to-one interviews were conducted face-to-face in a research room available at RBHT (*n* = 6), a vacant consultancy room (*n* = 6) and via phone (*n* = 4). All interviews were audio-recorded and transcribed verbatim. Interview durations ranged between 15 and 85 min. **Data Analysis:** Five steps of Theoretical Domains Framework-based interviews.
[38] (Ivynian et al., 2020) Australia	To assess patient-related factors thought to impact care-seeking, and examine the role of previous healthcare experiences in decisions to seek or avoid professional care.	Care-seeking	**Design:** Exploratory sequential mixed methods, with qualitative phenomenological emphasis. **Sample:** Patients diagnosed with heart failure. 72 participants were recruited (Refer to Table 1). The mean age was 61 ± 15 years (range 21–91 years). Most participants were male (68%) and Caucasian (71%). Participants (*n* = 15) who completed interviews were mostly male (*n* = 10), and the mean age was slightly younger than the entire cohort (58 ± 10 vs. 61 ± 15 years). **Sample Size:** 72(quantitative),15(qualitative) **Sampling Technique:** Purposive **Data Collection:** 1. Participants completed a series of self-report questionnaires. 2. Semi-structured, in-depth interviews were conducted to elicit information about previous care-seeking experiences and factors contributing to decisions to seek or avoid professional care. Interviews lasted 31–95 min in duration, were audio-recorded, and transcribed verbatim. **Data Analysis:** Qualitative data were analyzed using interpretative phenomenological analysis and interpreted in the context of quantitative findings.
[39] (Koontalay, Botti, & Hutchinson, 2024) Thailand	To explore the cognitive representations and emotional responses to living with chronic heart failure of people receiving limited community disease management.	Not provided	**Design:** Descriptive qualitative **Sample:** 20 hospitalized adults with CHF (16 men, 4 women; aged 42–78 years; duration of CHF: 1–30 years) in Thailand. **Sample Size:** 20 **Sampling Technique:** Purposive **Data Collection:** Semi-structured face-to-face interviews (30–45 min), conducted in Thai, audio-recorded, and translated to English. **Data Analysis:** Thematic content analysis (aligned with Leventhal’s model), reflexive thematic analysis for emotional responses.
[40] (Neumann et al., 2024) Germany, Ireland, The Netherlands, United Kingdom	To explore HF patients’ expectations, experiences, and usage behaviors regarding a self-care app (DoctorME), evaluating its role as a digital health tool in chronic HF management.	Not provided	**Design:** Longitudinal qualitative **Sample:** 83 HF patients (initial: 38; post: 45; male: initial 84%/post 78%; aged 60–69; NYHA Class I predominant). **Sample Size:** 83 **Sampling Technique:** Purposive maximum variation (age, gender, HF severity). **Data Collection:** Semi-structured interviews (mean duration: initial 33 min/post 23 min; face-to-face/phone/virtual; multilingual transcription). **Data Analysis:** Qualitative content analysis (Mayring’s approach), deductive-inductive coding (MAXQDA); inter-coder agreement.
[41] (Chai et al., 2024) USA	To explore the facilitators and barriers to using DPS technology to monitor pharmacotherapy adherence among patients with HF.	Not provided	**Design:** Mixed methods study (qualitative interviews + quantitative assessments). Qualitative design not stated. **Sample:** 20 HF patients (11 female, 9 males; mean age 68 years) **Sample Size:** 20 **Sampling Technique:** Convenience **Data collection:** Audio-recorded interviews (23–64 min) and Quantitative surveys. **Data Analysis:** Thematic analysis
[42] (Dickens, Dickson, & Piano, 2019) USA	To describe the influence of stress and social determinants of health on self-care in patients with HF who have low SES.	Social determinants of health	**Design:** Mixed-methods (concurrent embedded design); qualitative design not stated **Sample:** 35 adults (17 women, 18 men; 91.4% African American; age 36–89; low SES/homeless) **Sample Size:** 35 **Sampling Technique:** Purposive **Data Collection:** 1. Participants completed a series of self-report questionnaires. 2. Semi-structured face-to-face interviews (~90 min) **Data Analysis:** Content analysis (inductive + deductive).
[43] (Son, Oh, & Kim, 2020) South Korea	To explore heart failure patients’ needs and perspectives for using mobile health technology at home before developing a mobile phone-based heart failure self-care intervention	Mobile health	**Design:** Descriptive qualitative **Sample:** 20 adults (8 women, 12 men; age 30–80+) with chronic HF (NYHA Class I–II). **Sample Size:** 20 **Sampling Technique:** Purposive **Data Collection:** Semi-structured face-to-face interviews (average 100 min) in patients’ homes/outpatient clinics. **Data Analysis:** Content analysis
[44] (Trenta et al., 2021) Italy	To explore and describe the experience of self-care in adults with a retro-auricular left ventricular assist device (LVAD).	Self-care, self-care self-efficacy	**Design:** Interpretive description **Sample:** 9 men, 1 woman (ages 54–79; predominantly older Italian men with retro-auricular LVADs) **Sample Size:** 10 **Sampling technique:** Purposive **Data Collection:** Face-to-face semi-structured interviews and field notes **Data Analysis:** Interpretive Description
[45] (Johnston et al., 2022) Irealand	To design and evaluate a digital health tool (DHT) using human-centered design (HCD) to promote effective self-care in heart failure (HF) patients.	Not provided	**Design:** Mixed methods (HCD framework: empathize, ideate, design, develop, test). Qualitative design not stated. **Sample:** 19 patients, 6 women and 13 men, age range: 36–84 (study 1: interviews); 9 patients, 4 women and 5 men, age range: 54–91 (study 2: testing) **Sample Size:** 19 (qualitative), 9 (pilot) **Sampling Technique:** Purposeful (study 1), convenience (study 2) **Data Collection:** Semi-structured interviews, Fitbit activity trackers, smart scales, mobile app usage data **Data Analysis:** Thematic analysis (Braun & Clarke), quantitative adherence metrics (SUS, WTMS, CRS scores).
[46] (Myers et al., 2020) USA	To explore perceptions and motivations of HF patients who transitioned from non-adherence to adherence in self-care behaviors.	Perception, motivation	**Design:** Qualitative descriptive **Sample:** 8 HF patients (7 men, 1 woman; age 60–90; 5 Caucasian, 3 other ethnicities; 7 with HFrEF, all NYHA Class III) **Sample Size:** 8 **Sampling Technique:** Purposive **Data collection:** Semi-structured interviews (60 min, audio-recorded) **Data Analysis:** Braun & Clarke’s 6-step thematic analysis
[47] (Surikova et al., 2020) Canada	To identify cultural and gender considerations that might present intervention opportunities in order to improve self-care adherence and quality of life among chronic heart failure (CHF) patients.	Chronic heart failure	**Design:** Qualitative descriptive **Sample:** 30 CHF patients (67% male; 8 Black, 9 Chinese, 6 South Asian, 7 Caucasian). **Sample Size:** 30 **Sampling Technique:** Purposive **Data Collection:** Semi-structured interviews (60 min) on CHF experiences, lifestyle impact, and QoL **Data Analysis:** Thematic content analysis using NVivo 11.
[48] (Guidotti et al., 2022) Italy	To explore whether chronic heart failure (CHF) patients’ adherence to pharmaceutical therapies and self-care behavior recommendations changes across their care pathway and time, using Patient-Reported Outcome Measures (PROMs).	Adherence to long-term therapy	**Design:** Longitudinal mixed-methods (quantitative PROMs + qualitative open-text responses). Qualitative design not stated. **Sample:** CHF patients (75.86% male, mean age 71 ± 11 years, 64.94% retired) **Sample Size:** Baseline: 174; Follow-ups: 151 (1 month), 130 (7 months), 122 (12 months). **Sampling Technique:** Purposive **Data Collection:** Digital surveys at baseline and 1/7/12 months post-discharge. 1. Quantitative: SCHFI, KCCQ-12 (health status), PREMs (experience). 2. Qualitative: Open-ended questions on care experiences. **Data Analysis:** 1. Quantitative: Chi-Square, *t*-tests, multivariate regression (STATA15). 2. Qualitative: Thematic analysis (NVivo10^®^), word frequency coding.
[49] (Seckin et al., 2024) Turkey	To explore the experiences of Turkish individuals with heart failure, focusing on breathlessness symptoms, self-management strategies, and socio-cultural-behavioral contexts.	Not provided	**Design:** Qualitative descriptive **Sample:** 20 Turkish adults with self-reported heart failure and breathlessness (11 women, 9 men; mean age 55.5 ± 16.2 years). **Sample Size:** 20 **Sampling Technique:** Purposive, snowball **Data Collection:** Semi-structured interviews (11 face-to-face, 6 telephone, 3 email, 21–65 min). Audio-recorded, transcribed, and translated (Turkish to English) **Data Analysis:** Braun & Clarke’s 6-step thematic analysis
[50] (Radhakrishnan et al., 2020) USA	To explore perceptions and expectations of older adults with heart failure (HF) regarding sensor-controlled digital games (SCDGs) for HF self-management, focusing on personalization, usability, and motivational features.	Not provided	**Design:** Qualitative descriptive **Sample:** 15 patients with HF (53% women; age range, 53–90 years; 60% white). **Sample Size:** 15 **Sampling Technique:** Purposive **Data Collection:** Semi-structured interviews (30–45 min) in clinic or participant’s home. **Data Analysis:** Thematic analysis: inductive coding (Microsoft Word/Excel).
[51] (Celano et al., 2020) USA	To explore positive psychological constructs in heart failure (HF) patients and their perceived links to health behavior adherence	Not provided	**Design:** Qualitative descriptive **Sample:** 30 patients with HF (23 women, 7 men; Mean age: 67 years (SD 13.1)). **Sample Size:** 30 participants (baseline); 25 completed 3-month follow-up. **Sampling Technique:** Purposive **Data Collection:** 1. Baseline: Semi-structured interviews (20 in-person, 10 via phone). 2. Follow-up: Phone interviews at 3 months. Audio-recorded, transcribed, field notes. **Data Analysis**: Directed content analysis
[52] (Hall et al., 2014) USA	To explore heart failure (HF) patients’ perceptions and current use of technology for managing HF symptoms, and identify barriers/facilitators to technology adoption.	Not provided	**Design:** Descriptive qualitative **Sample:** 15 adults with an age range of 45–82 years; mean:64.43 years (SD 10.28). **Sample Size:** 15 **Sampling Technique:** Convenience **Data Collection:** Semi-structured interviews (10–30 min). Audio-recorded, transcribed verbatim. **Data Analysis:** Constant comparative method
[53] (Carroll, Hahn, & Grady, 2022) USA	To evaluate research engagement and experiences of adults with mechanical circulatory support (MCS) pre- and post-LVAD implant, focusing on health-related quality of life (HRQOL) assessments.	Not provided	**Design:** Mixed-methods (quantitative structured interviews + qualitative open-ended responses). Qualitative design not stated. **Sample:** Patients with advanced heart failure receiving or awaiting LVAD implantation (78% male, mean age ~60) **Sample Size:** 687 **Sampling Technique:** Convenience **Data Collection:** 1. Quantitative: Debrief interview with structured items (e.g., Likert scales on difficulty and experience) 2. Qualitative: Open-ended questions on questionnaire experiences and suggestions **Data Analysis:** 1. Quantitative: Descriptive stats, chi-square, *t*-tests. 2. Qualitative: Thematic analysis with coding agreement (kappa > 0.70)

**Table 3:** Thematic synthesis.

Analytical Theme	Supporting Studies	Descriptive Theme	Codes
**1. Adaptive and Technology-Supported Self-Management in Daily Life**	[35,36,37,38,40,43,44,45,47,48,49,50,51,52]	**Recognizing and Responding to Symptoms**	Developing Symptom Awareness Anticipating and Preparing for Symptoms Cultural Framing of Symptom Interpretation
		**Activity, Environment, and Routine Modification**	Strategically Conserving Energy for Daily Functioning Modifying the Physical Environment to Reduce Strain Integrating Culturally Meaningful Activities into Routines
		**Monitoring and Adjusting Behavior**	Employing Self-Monitoring Tools to Track Health Status Modifying Daily Activities in Response to Monitoring Data Building Confidence and Acceptance in Technology Use
		**Coping with Challenges and Temptations**	Actively Avoiding Foods that Exacerbate Symptoms Replacing Risky Choices with Health-Supportive Alternatives Balancing Occasional Indulgence with Corrective Self-Management
		**Technology as a Self-Care Facilitator**	Leveraging Digital Reminders to Support Adherence Utilizing Digital Platforms for Tracking and Achieving Health Goals Adapting Technology to Align with Cultural Values and Practices
		**Barriers to Technology Use**	Limited Digital Literacy Restricting Effective Technology Use Reliance on In-Person Communication over Digital Alternatives Skepticism Toward the Usefulness and Reliability of Technology
**2. Emotional and Psychological Dimensions of Self-Care**	[35,36,37,38,39,41,42,47,49]	**Emotional Impact of Symptoms**	Fear of Health Deterioration and Mortality Anxiety Driven by the Unpredictability of Symptoms Cultural and Spiritual Interpretations Shaping Emotional Responses
		**Identity and Role Changes**	Emotional Distress from Reduced Autonomy Emotional Impact of Being Unable to Fulfill Culturally Significant Roles Perceived Burden and Declining Sense of Social Value
**3. Social, Cultural, and Environmental Influences on Self-Care**	[37,38,39,40,41,43,44,45,46,48,49,50,53]	**Support and Guidance from Healthcare Providers**	Motivation through Trusted Professional Support Building Confidence through Professional Reassurance Enhancing Self-Care through Culturally Sensitive Guidance
		**Social Support and Companionship**	Strengthening Motivation through Shared Physical Activity Emotional and Practical Reinforcement from Social Networks Restrictive Effects of Overprotective Caregiving
		**Cultural Contexts in Self-Care**	Dietary Choices Shaped by Social Expectations and Obligations Managing Self-Care Amid Festive and Seasonal Food Traditions Respect for Elders and Traditional Practices Guiding Dietary Behaviors
		**Motivation through Emotional and Cultural Connections**	Family Care as a Source of Motivation for Self-Care Safeguarding Children’s Well-Being as a Driver of Self-Care Self-Care as Fulfillment of Cultural and Familial Responsibilities

## Data Availability

No new data were created or analyzed in this study.

## Supplementary Materials

The following supporting information can be downloaded at: https://www.mdpi.com/article/10.3390/jcm15176723/s1. Table S1: PRISMA 2020 checklist. Table S2: Full search strategy. Table S3: List of excluded studies.

## Abbreviations

HF	Heart Failure
CHF	Chronic Heart Failure
SCHFI	Self-Care of Heart Failure Index
CASP	Critical Appraisal Skills Program

## References

[1] Aghajanloo A., Negarandeh R., Janani L., Tanha K., Hoseini-Esfidarjani S. (2021). Self-care status in patients with heart failure: Systematic review and meta-analysis. Nurs. Open.

[2] Seid S.S., Amendoeira J., Ferreira M.R. (2023). Self-care and quality of life among adult patients with heart failure: Scoping review. SAGE Open Nurs..

[3] Da Costa Ferreira Oberfrank N., D’Agostino F., Watkinson E., Takao Lopes C., D’Angelo D., Sanson G. (2025). Effectiveness of Interventions to Improve Heart Failure Self-care: An Umbrella Review of Systematic Reviews. J. Cardiovasc. Nurs..

[4] Kleman C., Turrise S., Winslow H., Alzaghari O., Lutz B.J. (2024). Individual and systems-related factors associated with heart failure self-care: A systematic review. BMC Nurs..

[5] Harkness K., Spaling M.A., Currie K., Strachan P.H., Clark A.M. (2015). A Systematic Review of Patient Heart Failure Self-care Strategies. J. Cardiovasc. Nurs..

[6] Lovell J., Pham T., Noaman S.Q., Davis M.C., Johnson M., Ibrahim J.E. (2019). Self-management of heart failure in dementia and cognitive impairment: A systematic review. BMC Cardiovasc. Disord..

[7] Spaling M.A., Currie K., Strachan P.H., Harkness K., Clark A.M. (2015). Improving support for heart failure patients: A systematic review to understand patients’ perspectives on self-care. J. Adv. Nurs..

[8] Liu S., Li J., Wan Dyuan Li R., Qu Z., Hu Y., Liu J. (2022). Effectiveness of eHealth Self-management Interventions in Patients With Heart Failure: Systematic Review and Meta-analysis. J. Med. Internet Res..

[9] Olofsson S., Josephsson H., Lundvall M., Lundgren P., Wireklint Sundström B. (2025). Patient participation in self-monitoring regarding healthcare of heart failure: An integrated systematic review. BMC Prim. Care.

[10] Umeh C.A., Torbela A., Saigal S., Kaur H., Kazourra S., Gupta R., Shah S. (2022). Telemonitoring in heart failure patients: Systematic review and meta-analysis of randomized controlled trials. World J. Cardiol..

[11] Wang C. (2024). Role of Telemedicine Intervention in the Treatment of Patients with Chronic Heart Failure: A Systematic Review and Meta-analysis. Anatol. J. Cardiol..

[12] Bekele F., Tafese L., Demsash A.W., Tesfaye H., Labata B.G., Fekadu G. (2023). Adherence to self-care practices and associated factors among heart failure patients in Ethiopia: A systematic review and meta-analysis. PLoS ONE.

[13] Bahrodi P.S., Safa A., Ajorpaz N.M., Avanji F.S.I. (2024). Heart failure patients’ experiences of self-care neglect: A content analysis. BMC Cardiovasc. Disord..

[14] Longhini J., Gauthier K., Konradsen H., Palese A., Kabir Z.N., Waldréus N. (2025). The effectiveness of nursing interventions to improve self-care for patients with heart failure at home: A systematic review and meta-analysis. BMC Nurs..

[15] Kyriakou M., Samara A., Philippou K., Lakatamitou I., Lambrinou E. (2021). A qualitative meta-synthesis of patients with heart failure perceived needs. Rev. Cardiovasc. Med..

[16] Li Y., Xiong X., Wang H., Chen L., Wu R., Zhang M., Xiang Q., Liu S., Chen H., Xiao D. (2025). Positive psychological experiences in chronic heart failure: A qualitative meta-synthesis. J. Adv. Nurs..

[17] Sandelowski M., Docherty S., Emden C. (1997). Focus on qualitative methods. Qualitative metasynthesis: Issues and techniques. Res. Nurs. Health.

[18] Thorne S., Jensen L., Kearney M.H., Noblit G., Sandelowski M. (2004). Qualitative metasynthesis: Reflections on methodological orientation and ideological agenda. Qual. Health Res..

[19] Finfgeld-Connett D., Johnson E.D. (2013). Literature search strategies for conducting knowledge-building and theory-generating qualitative systematic reviews. J. Adv. Nurs..

[20] Lachal J., Revah-Levy A., Orri M., Moro M.R. (2017). Metasynthesis: An Original Method to Synthesize Qualitative Literature in Psychiatry. Front. Psychiatry.

[21] Thorne S. (2017). Metasynthetic Madness: What Kind of Monster Have We Created?. Qual. Health Res..

[22] Sim J., Mengshoel A.M. (2023). Metasynthesis: Issues of empirical and theoretical context. Qual. Quant..

[23] Page M.J., McKenzie J.E., Bossuyt P.M., Boutron I., Hoffmann T.C., Mulrow C.D., Shamseer L., Tetzlaff J.M., Akl E.A., Brennan S.E. (2021). The PRISMA 2020 statement: An updated guideline for reporting systematic reviews. BMJ.

[24] Rossi L.P., Granger B.B., Bruckel J.T., Crabbe D.L., Graven L.J., Newlin K.S., Streur M.M., Vadiveloo M.K., Walton-Moss B.J., Warden B.A. (2023). Person-centered models for cardiovascular care: A review of the evidence: A scientific statement from the American Heart Association. Circulation.

[25] Goldfarb M.J., Saylor M.A., Bozkurt B., Code J., Di Palo K.E., Durante A., Flanary K., Masterson Creber R., Ogunniyi M.O., Rodriguez F. (2024). Patient-centered adult cardiovascular care: A scientific statement from the American Heart Association. Circulation.

[26] Gusenbauer M., Gauster S.P. (2025). How to search for literature in systematic reviews and meta-analyses: A comprehensive step-by-step guide. Technol. Forecast. Soc. Change.

[27] Aloudah N.M. (2022). Qualitative research in the Arabic language. When should translations to English occur? A literature review. Explor. Res. Clin. Soc. Pharm..

[28] Neimann Rasmussen L., Montgomery P. (2018). The prevalence of and factors associated with inclusion of non-English language studies in Campbell systematic reviews: A survey and meta-epidemiological study. Syst. Rev..

[29] Morrison A., Polisena J., Husereau D., Moulton K., Clark M., Fiander M., Mierzwinski-Urban M., Clifford T., Hutton B., Rabb D. (2012). The effect of English-language restriction on systematic review-based meta-analyses: A systematic review of empirical studies. Int. J. Technol. Assess. Health Care.

[30] Amano T., Berdejo-Espinola V. (2025). Language barriers in conservation: Consequences and solutions. Trends Ecol. Evol..

[31] Dobrescu A.I., Nussbaumer-Streit B., Klerings I., Wagner G., Persad E., Sommer I., Herkner H., Gartlehner G. (2021). Restricting evidence syntheses of interventions to English-language publications is a viable methodological shortcut for most medical topics: A systematic review. J. Clin. Epidemiol..

[32] Helbach J., Pieper D., Mathes T., Rombey T., Zeeb H., Allers K., Hoffmann F. (2022). Restrictions and their reporting in systematic reviews of effectiveness: An observational study. BMC Med. Res. Methodol..

[33] Meline T. (2006). Selecting Studies for Systemic Review: Inclusion and Exclusion Criteria. Contemp. Issues Commun. Sci. Disord..

[34] Hannah A.L., David P.F., Joanna M.B. (2020). Optimising-the-value-of-the-critical-appraisal-skills-programme-casp-tool-for-quality-appraisal-in.pdf. Res. Methods Med. Health Sci..

[35] Walthall H., Jenkinson C., Boulton M. (2017). Living with breathlessness in chronic heart failure: A qualitative study. J. Clin. Nurs..

[36] Chew H.S.J., Sim K.L.D., Cao X., Chair S.Y. (2019). Motivation, Challenges and Self-Regulation in Heart Failure Self-Care: A Theory-Driven Qualitative Study. Int. J. Behav. Med..

[37] Amirova A., Lucas R., Cowie M.R., Haddad M. (2022). Perceived barriers and enablers influencing physical activity in heart failure: A qualitative one-to-one interview study. PLoS ONE.

[38] Ivynian S.E., Ferguson C., Newton P.J., DiGiacomo M. (2020). Factors influencing care-seeking delay or avoidance of heart failure management: A mixed-methods study. Int. J. Nurs. Stud..

[39] Koontalay A., Botti M., Hutchinson A. (2024). Illness perceptions of people living with chronic heart failure and limited community disease management. J. Clin. Nurs..

[40] Neumann A., Steiner B., Verket M., Kanna N.D., Hill L., McNulty A., Boyne J.J., Murphy M., Maaser Y., Fitzsimons D. (2024). Patients’ expectations and experiences with the usage of a self-care application for heart failure: A qualitative interview study. Digit. Health.

[41] Chai P.R., Kaithamattam J.J., Chung M., Tom J.J., Goodman G.R., Hasdianda M.A., Carnes T.C., Vaduganathan M., Scirica B.M., Schnipper J.L. (2024). Formative perceptions of a digital pill system to measure adherence to heart failure pharmacotherapy: Mixed methods study. JMIR Cardio.

[42] Dickens C., Dickson V.V., Piano M.R. (2019). Perceived stress among patients with heart failure who have low socioeconomic status: A mixed-methods study. J. Cardiovasc. Nurs..

[43] Son Y.J., Oh S., Kim E.Y. (2020). Patients’ needs and perspectives for using mobile phone interventions to improve heart failure self-care: A qualitative study. J. Adv. Nurs..

[44] Trenta A.M., Luciani M., Moro M., Patella S., Di Mauro S., Vellone E., Ausili D. (2022). Self-care in adults with a retro-auricular left ventricular assist device: An interpretive description. Clin. Nurs. Res..

[45] Johnston W., Keogh A., Dickson J., Leslie S.J., Megyesi P., Connolly R., Burke D., Caulfield B. (2022). Human-Centred Design Of A Digital Health Tool To Promote Effective Self-Care In Heart Failure Patients: Mixed Methods Study. JMIR Form. Res..

[46] Myers S.L., Siegel E.O., Hyson D.A., Bidwell J.T. (2020). A qualitative study exploring the perceptions and motivations of patients with heart failure who transitioned from non-adherence to adherence. Heart Lung.

[47] Surikova J., Payne A., Miller K.L., Ravaei A., Nolan R.P. (2020). A cultural and gender-based approach to understanding patient adjustment to chronic heart failure. Health Qual. Life Outcomes.

[48] Guidotti E., Pennucci F., Valleggi A., De Rosis S., Passino C. (2022). A longitudinal assessment of chronic care pathways in real-life: Self-care and outcomes of chronic heart failure patients in Tuscany. BMC Health Serv. Res..

[49] Seckin M., Petrie M.C., Stewart S., Johnston B.M. (2024). Descriptive qualitative study of breathlessness and its management of Turkish individuals with self-reported heart failure. BMJ Open.

[50] Radhakrishnan K., Baranowski T., O’Hair M., Fournier C.A., Spranger C.B., Kim M.T. (2020). Personalizing sensor-controlled digital gaming to self-management needs of older adults with heart failure: A qualitative study. Games Health J..

[51] Celano C.M., Beale E.E., Freedman M.E., Mastromauro C.A., Feig E.H., Park E.R., Huffman J.C. (2020). Positive psychological constructs and health behavior adherence in heart failure: A qualitative research study. Nurs. Health Sci..

[52] Hall A.K., Dodd V., Harris A., McArthur K., Dacso C., Colton L.M. (2014). Heart failure patients’ perceptions and use of technology to manage disease symptoms. Telemed. e-Health.

[53] Carroll A.J., Hahn E.A., Grady K.L. (2022). Research engagement and experiences of patients pre- and post-implant of a left ventricular assist device from the mechanical circulatory support measures of adjustment and quality of life (MCS A-QOL) study. Qual. Life Res..

[54] Younas A., Ali P. (2021). Five tips for developing useful literature summary tables for writing review articles. Evid.-Based Nurs..

[55] Thomas J., Harden A. (2008). Methods for the thematic synthesis of qualitative research in systematic reviews. BMC Med. Res. Methodol..

[56] Aimo A., Teresi L., Castiglione V., Picerni A.L., Niccolai M., Severino S., Agazio A., Carnevale Baraglia A., Obici L., Palladini G. (2024). Patient-reported outcome measures for transthyretin cardiac amyloidosis: The ITALY study. Amyloid.

