# Supplementary material for: Self-Care Experiences of Individuals with Heart Failure: A Systematic Review and Metasynthesis
Source: J Clin Med. 2026 Aug 29;15(17):6723. doi: 10.3390/jcm15176723 (PMC13566109; doi:10.3390/jcm15176723)
Supplement: Supplementary file 1 [file jcm-15-06723-s001.zip › Table S2 Full search strategy.pdf]

Table S2 Full search strategy

PubMed string
(self-care OR self-management) AND heart failure AND (behavior OR health risk behaviors) AND (in-hospital days OR quality of life OR mortality OR rehospitalization OR health care costs) AND qualitative research Filters: from 2014 – 2025
Scopus string
(ALL (self-care OR self-management) AND ALL (heart AND failure) AND ALL (behavior OR health AND risk AND behaviors) AND ALL (in-hospital AND days OR quality AND of AND life OR mortality OR rehospitalization OR health AND care AND costs) AND ALL (qualitative AND research)) AND PUBYEAR > 2013 AND PUBYEAR < 2025 AND (LIMIT-TO (LANGUAGE , "English"))
Web of Science string
self-care OR self-management (All Fields) AND heart failure (All Fields) AND behavior OR health risk behaviors (All Fields) AND in-hospital days OR quality of life OR mortality OR rehospitalization OR health care costs (All Fields) AND qualitative research (All Fields) and 2025 or 2024 or 2023or 2022or 2021 or 2020 or 2018 or 2022 or 2015 or 2017 or 2024 or 2014 or 2016 or 2019 (Publication Years)
ProQuest Central
(self-care OR self-management) AND heart failure AND (behavior OR health risk behaviors) AND (in-hospital days OR quality of life OR mortality OR rehospitalization OR health care costs) AND qualitative research Filters: from 2014 – 2025
DANS – Data Archiving and Networked Services (OpenGrey)
(self-care OR self-management) AND heart failure AND (behavior OR health risk behaviors) AND (in-hospital days OR quality of life OR mortality OR rehospitalization OR health care costs) AND qualitative research Filters: from 2014 – 2025
OAIster

(ti: (self-care OR self-management) AND heart failure AND (ti: (behavior OR health risk behaviors) AND (ti: (in-hospital days OR quality of life OR mortality OR rehospitalization OR health care costs) AND (ti: qualitative research Filters: from 2014 – 2025
