# Supplementary material for: Self-Care Experiences of Individuals with Heart Failure: A Systematic Review and Metasynthesis
Source: J Clin Med. 2026 Aug 29;15(17):6723. doi: 10.3390/jcm15176723 (PMC13566109; doi:10.3390/jcm15176723)
Supplement: Supplementary file 1 [file jcm-15-06723-s001.zip › Table S3 List of excluded studies.pdf]

Table S3 List of excluded studies

Wrong outcome	• Radhakrishnan, K., Allen, C., DeMain, A. S., & Park, L. (2021). Impact of COVID-19 on heart failure self-care: a qualitative study. *Journal of Cardiovascular Nursing*, *36*(6), 609-617.<br>• Sousa, J. P., & Santos, M. (2019). Symptom management and hospital readmission in heart failure patients: a qualitative study from Portugal. *Critical care nursing quarterly*, *42*(1), 81-88.<br>• Liljeroos, M., Ågren, S., Jaarsma, T., & Stromberg, A. (2017). Dialogues between nurses, patients with heart failure and their partners during a dyadic psychoeducational intervention: a qualitative study. *BMJ open*, *7*(12), e018236.<br>• Pedersen, S. S., Schmidt, T., Skovbakke, S. J., Wiil, U. K., Egstrup, K., Smolderen, K. G., & Spertus, J. A. (2017). A personalized and interactive web-based health care innovation to advance the quality of life and care of patients with heart failure (ACQUIRE-HF): a mixed methods feasibility study. *JMIR Research Protocols*, *6*(5), e7110.<br>• Guo, X., Gu, X., Jiang, J., Li, H., Duan, R., Zhang, Y., ... & Chen, F. (2019). A hospital-community-family–based telehealth program for patients with chronic heart failure: single-arm, prospective feasibility study. *JMIR mHealth and uHealth*, *7*(12), e13229.<br>• Cowie, M. R., Anker, S. D., Cleland, J. G., Felker, G. M., Filippatos, G., Jaarsma, T., ... & López-Sendón, J. (2014). Improving care for patients with acute heart failure: before, during and after hospitalization. *ESC heart failure*, *1*(2), 110-145.

	• Segan, L., Nanayakkara, S., Mak, V., & Kaye, D. (2018). Enhancing self-care strategies in heart failure through patient-reported outcome measures. *Internal medicine journal*, *48*(8), 995-998.<br>• Charuel, E., Bernard, M., Vaillant Roussel, H., Cambon, B., Ménini, T., Lafarge, E., ... & Vorilhon, P. (2022). "I Can't Go Far": perceptions and experiences of heart failure patients regarding physical activity: a qualitative study using semistructured face-to-face interviews. *Health Education & Behavior*, *49*(1), 78-86.
Wrong study design	• Sano, M., & Majima, T. (2021). Development of a home-based nursing intervention model for patients with heart failure: a qualitative feasibility study. *INQUIRY: The Journal of Health Care Organization, Provision, and Financing*, *58*, 00469580211067448.<br>• Farmer, S. A., Magasi, S., Block, P., Whelen, M. J., Hansen, L. O., Bonow, R. O., ... & Grady, K. L. (2016, August). Patient, caregiver, and physician work in heart failure disease management: a qualitative study of issues that undermine wellness. In *Mayo Clinic Proceedings* (Vol. 91, No. 8, pp. 1056-1065). Elsevier.
Wrong population	• Buck, H. G., Hupcey, J., Wang, H. L., Fradley, M., Donovan, K. A., & Watach, A. (2018). Heart failure self-care within the context of patient and informal caregiver dyadic engagement: a mixed methods study. *Journal of Cardiovascular Nursing*, *33*(4), 384-391.<br>• Kimani KN, Murray SA, Grant L. Multidimensional needs of patients living and dying with heart failure in Kenya: A serial interview study. BMC Palliative Care. 2018;17(1):1–8.
